# Commentary: Reimagining Community Mental Health Care Services: Case Study of a Need Based Biopsychosocial Response Initiated During Pandemic

**DOI:** 10.3389/fpsyt.2022.859884

**Published:** 2022-05-27

**Authors:** J. Steven Lamberti

**Affiliations:** Department of Psychiatry, University of Rochester Medical Center, Rochester, NY, United States

**Keywords:** pandemic (COVID-19), severe mental disorder, schizophrenia, health service access, telehealth, community partnership and service, public mental health, foundation partnerships

As a professor at a Western medical center, I first want to acknowledge my inexperience with the Indian healthcare system as well as resource differences that exist between our care systems. Also, I work at the University of Rochester Medical Center (URMC), which gave birth to the biopsychosocial model and is the focus of this special issue. With these standpoints in mind, I offer the following commentary on Sunder's et al. recent article (1). I'll begin with some background about URMC followed by discussion of the coronavirus pandemic to provide context. Then I will discuss the exemplary work currently being done in Kerala, India along with our local efforts to address the pandemic. Lastly, I'll highlight potential advantages of Kerala's innovative approach to care.

## The Biopsychosocial Model

The biopsychosocial model was proposed in 1977 by Engel (2) who trained as an internist, in collaboration with psychiatrist Dr. John Romano (3). In contrast to the prevailing biomedical ethos of the time and its myopic focus on biological processes, Engle and Romano's theory provided conceptual links between the body, the mind, and society. Yet translating this conceptual framework into clinical practice has remained a challenge for mental health service delivery in the United States. A 2009 report by the National Alliance on Mental Illness (NAMI) rating overall quality of mental health services gave the United States a “D” grade (i.e., unacceptably poor) (4). One of NAMI's key recommendations was to better integrate mental and physical healthcare through co-location of medical and behavioral health professionals. Progress has since been made in service integration (5, 6), forging real-world healthcare bridges between the biological and psychological dimensions of the biopsychosocial model. The coronavirus pandemic, however, has recently revealed an alarming disconnect between healthcare and society in the United States.

## The Great Pandemic

The coronavirus pandemic has now accounted for more deaths in the United States than the Influenza Pandemic of 1918 (7), this despite advances in public health and widely available vaccines. Many in the United States refuse to be vaccinated or to wear masks despite the proven effectiveness of these strategies (8–10). In explaining this impasse, scholars have pointed to the role of social and cultural factors including politicization and media sensationalism (11, 12). These influences have led some to view public health practices as an affront to personal liberty, thus undermining trust in medical authorities and healthcare providers alike.

People with severe mental illness are among those now bearing the brunt of the pandemic (13). For example, individuals with schizophrenia have high rates of mortality following coronavirus infection (14) in addition to having poor access to healthcare services (15, 16). Lockdown strategies to contain the virus have threatened to further limit healthcare access both here in Rochester and in Kerala, India. In response, healthcare providers in both regions have worked to promote access to treatment for people with severe mental illness through telehealth strategies. Yet there have also been differences in how these communities have faced the challenge of delivering healthcare to their most vulnerable citizens in the midst of a deadly pandemic.

## Kerala, India

Prior to the pandemic, Kerala had developed a reputation for achieving good health outcomes despite having a low per capita income (17). With limited healthcare resources, Kerala's Mehac Foundation undertook a novel and highly efficient approach to care delivery that required broad and active community participation. Borrowing from the field of palliative care, the foundation implemented a flexible model of service delivery based on Public-Private-People Partnership (1). This approach utilized existing community resources including public governance organizations (e.g., panchayats), private organizations (e.g., non-governmental and corporate organizations), and—most notably—people (e.g., family members and volunteers). Getting all of these individuals and organizations to pull together required a shared sense of purpose as well as high levels of communication and cooperation. Mehac provided the necessary vision while using existing ties with governmental and volunteering agencies to access local healthcare professionals, to link patients to services and to ensure medication delivery. The foundation also made substantive efforts to supply education, food, and monetary support to those in need.

## Rochester, New York, USA

The URMC Department of Psychiatry is home to Strong Ties, an outpatient clinic for people with severe mental disorders. Prior to the pandemic, over 90% of services were delivered within the walls of our clinic. At the height of the pandemic, Strong Ties utilized telehealth services for 70% of all patient contacts. Live visits were generally limited to crisis intervention, new patients and those without telephone or computer access. Delivery of medications, clothing and food was conducted through a team of care managers and two assertive community treatment teams along with use of community pharmacies for prescriptions. Although these approaches ensured continuity of care, they did not necessarily build resilience among service recipients.

## Comments from Rochester to Kerala

Mehac's novel strategy of involving a wide-ranging coalition of agencies and individuals is consistent with current recommendations for optimizing continuity of care for people with severe mental illness during the pandemic (18). However, Mehac's implementation is likely to have significant benefits beyond simply maintaining continuity of healthcare. In particular, their emphasis on community engagement may *improve* mental health by directly addressing social determinants including poverty and lack of health literacy (19, 20). Also, research has suggested that being negatively judged by others is among the most harmful stressors for people with severe mental illness (21). Such stress within family settings is strongly associated with increased rates of psychotic relapse and hospitalization (22). Mehac's efforts to educate, support and empower “family as the unit of care” are therefore likely to reduce the need for psychiatric hospitalization by reducing stress and stigma within the home. Lastly, engaging a broad social fabric of community stakeholders is likely to build confidence and trust in healthcare professionals as leaders in the fight against COVID-19.

Sunder et al. (1) have acknowledged that a formal evaluation of Mehac's approach has yet to be conducted, and we look forward to that possibility. Until then, I commend my Indian colleagues for their exemplary leadership in addressing the social dimension of the biopsychosocial model.

## Author Contributions

The author confirms being the sole contributor of this work and has approved it for publication.

## Conflict of Interest

The author declares that the research was conducted in the absence of any commercial or financial relationships that could be construed as a potential conflict of interest.

## Publisher's Note

All claims expressed in this article are solely those of the authors and do not necessarily represent those of their affiliated organizations, or those of the publisher, the editors and the reviewers. Any product that may be evaluated in this article, or claim that may be made by its manufacturer, is not guaranteed or endorsed by the publisher.
